# Psychopathology of Young Terrorist Offenders, and the Interaction With Ideology and Grievances

**DOI:** 10.3389/fpsyt.2022.801751

**Published:** 2022-03-08

**Authors:** Nils Duits, Daphne L. Alberda, Maaike Kempes

**Affiliations:** Department of Science and Education, Netherlands Institute of Psychiatry and Psychology, Utrecht, Netherlands

**Keywords:** convicted terrorists, young terrorist offenders, psychopathology, ideology, grievances, European Database, forensic psychiatry and psychology

## Abstract

Psychopathology might be a risk factor for terrorist offending as it is for violent offending. Therefore, we examined the prevalence of psychopathology in young and adult Jihadist terrorist offenders on the basis of primary source judicial information and forensic mental health reports with the European Database of convicted Terrorist offenders (EDT). We hypothesised that psychopathology might be associated with ideological risk factors, and that these associations might be different for young and adult terrorist offenders. Therefore, we examined whether and to what extent psychopathology is related to a violent ideology, to grievances and anger about perceived injustice. We investigated whether this differs among 120 adult and 46 juvenile terrorist offenders. We found that most adult and young Jihadist terrorist offenders with a forensic mental health report had psychopathological problems. Most frequently found were symptoms and traits of intellectual disability disorders, depressive disorders, psychotic/schizophrenic disorders, substance use disorders, and personality disorders. Most frequently found clinically relevant personality traits were problems with relationships, poor regulation of aggression, feelings of anger, and paranoid feelings. We found some first indications for a positive association between psychopathology and grievances and anger about perceived injustice. In the young terrorist offenders with depressive symptoms, grievances about perceived injustice were more often present than in young terrorist offenders without these symptoms. In adult terrorist offenders it was found that grievances about perceived injustice and the anger were related to cluster B personality traits. In addition, in both young and adult terrorist offenders expressed grievances about perceived injustice were related to problems with relationships. Further research into psychopathology in terrorist offenders seems necessary with larger groups of adolescents and adults in relation to ideological, personal and contextual risk factors and how these factors relate to different terrorist acts. This may lead to more knowledge about engagement into terrorism and possible disengagement from terrorism. It may also lead to the inclusion of psychopathology into violent extremism risk assessment tools.

## Introduction

Countering terrorism is a global security challenge (1). Although many definitions and types of terrorism exist, one can define terrorism in a more general way as the threat, preparation, or perpetration of serious violence based on ideological motives against people, or deeds aimed at causing socially disruptive material damage with the goal to cause social change, to instil fear among the population or to influence political decision-making (2–4). Therefore, being a member of or participating in a terrorist organisation, threatening with terrorist attacks, recruiting, and financing terrorism are also considered terrorist offences.

To counter terrorism more effectively several authors, stress the need of an evidence-based approach to terrorist engagement, risk assessment and risk management with more knowledge about personal and contextual risk factors (5–7). Moreover, research is needed into the relationship between psychopathology and commitment to violent extremist ideologies in juveniles (8) and how psychopathology relates to “grievance-fueled targeted violence” in relation to other personal and contextual risk factors (9).

From 2015 we developed the European Database of convicted Terrorist offenders (EDT). This research database makes it possible to study specific risk and protective factors in convicted terrorist offenders. A unique aspect of the EDT is that psychopathology and other risk factors have been extensively operationalized and collected on primary source data, among which forensic mental health assessments (10). In this paper we examine to what extent terrorist offenders show psychopathology, how this is related to ideological beliefs and grievances, and whether this differs between adult and juvenile terrorist offenders.

Several authors showed in their studies that psychopathology is a risk factor for interpersonal violence in adult and young offenders, and that it has to be considered in conjunction with other risk factors (11–21). This is also reflected in structured professional judgement models of violent risk assessment and risk management, i.e., risk-based interventions used in adult offenders (11, 22) and juvenile offenders (20, 23–25). Clinical professionals must weigh the relevance of risk factors in relation to an individual's past violence, a person's decision to act violently in the future, the influence or impairment on a person's ability to use non-violent problem solving for example through psychopathology, and in responsivity or in (non)compliance with risk management (11, 22).

Although psychopathology evidently plays a role in general violent offending and consequently in its risk assessment and management, the evidence about the role of psychopathology in terrorist offending is less clear (26). Most evidence is restricted to lone actor adult terrorist offenders who may have a higher prevalence of schizophrenia, delusional disorder and autism spectrum disorders (27–29). In addition, research on psychopathology in terrorist offenders is generally aimed at revealing prevalence of mental disorders and not at its traits and symptoms (27–29). Moreover, in forensic mental health assessments of adults, forensic professionals often focus on clinically significant traits and symptoms of mental disorders rather than mental disorders *per se*. This is also due to comorbidity of mental disorders, and a lack of information because suspects or convicts withhold information. This focus on relevant clinically significant traits and symptoms, proves to be important in explaining delinquent and terrorist behaviour in relation to personal, social, and contextual circumstances. A good symptom-level description of psychopathology and its complex relationship with other factors, while also exploring relevant symptoms at the time of the terrorist offence, is important for improving clinical assessments and empirical research in terrorism (9, 30).

Psychopathology will seldom be a causal factor in the engagement of violent extremism and terrorism. However, specific psychological functions and processes, particularly maladaptive ones, might be relevant for understanding a person's pathway into and through violent extremism and terrorist activity (5, 8, 30). Commitment to an ideology that justifies the use of violence, and grievances about perceived injustice, and the anger or outrage in response to perceived injustice are specific for violent extremism and mostly for terrorism (5, 7, 31–34). These factors are also found to be important indicators in structured professional risk assessment tools, such as the Violent Extremism Risk Assessment (VERA-2R) and TRAP-18 (35–37). On the other hand, violent ideology or extreme overvalued beliefs might be diagnosed as a psychotic disorder as was manifested in the first psychiatric assessment in the court case of Breivik (38, 39).

Youth do join terrorist groups, also due to active engagement with online social media, and radicalised networks, personal grievances and triggering events but little is known about the role of psychopathology (40). A developmentally informed understanding of how adolescents and young adults become involved in violent extremism and terrorism is considered critical, in combination with the exploration of the possible relationship with mental health symptoms (8). Specifically, since childhood risk factors and adolescent conduct problems might be risk factors for adult involvement in terrorist acts (41). In addition, in forensic mental health assessments of adolescents a developmental psychopathological perspective is required in relation to their development and neuromaturation, focusing on traits and symptoms of possible dysfunction in various settings (42–44).

Grievances and anger about perceived injustice and a hostile attribution style are also symptoms and traits of psychopathology such as in antisocial youth, personality disorders, and in psychosis (45–47). It could therefore be argued that psychopathology in combination with perceived grievances and commitment to a violent ideology might be interrelated and/or may make individuals more prone to violent extremism and terrorism. Therefore, it seems appropriate to investigate what behavioural health symptoms exist in youth and how they may be related to violent extremist ideologies (8, 40).

Furthermore, it may be relevant to distinguish between adults and adolescents. In adolescence many psychological and behavioural health problems develop in combination with cognitive issues of self-control, planning and behavioural inhibition that are relevant at the intersection of adolescent development and adulthood, and this is also reflected in the differences between juvenile and adult offenders (43, 44). These problems may also be relevant for young terrorist offenders. Also, juveniles may be more likely to be convinced of a belief system and, without fully evaluating it or exploring alternatives, jumping to conclusions (46). This combination of psychological, behavioural health and cognitive problems and jumping to conclusions in relation to a belief system may also be relevant for young terrorist offenders. Therefore, it would be interesting to discern what behavioural health problems or symptoms are common among juvenile and adult terrorist offenders and what relationship—if any—exists between psychopathology with the commitment to a violent extremist ideology and perceived grievances (8, 40).

In this paper we examine if and to what extent terrorist offenders have psychopathology including clinical relevant symptoms and traits, and whether this differs between adult and juvenile terrorist offenders. We also examine whether psychopathology is related to violent extremist ideology, grievances, and the anger about perceived injustice, and whether this differs between adult and young terrorist offenders.

## Materials and Methods

### Definition of Terrorism

In the literature many definitions and types of terrorism exist, with a tendency to equate radicalism with extremism and terrorism (3, 4, 48). In this article we us the following definition for terrorism: the threat, preparation, or perpetration of serious violence based on ideological motives against people, or deeds aimed at causing socially disruptive material damage with the goal to cause social change, to instil fear among the population or to influence political decision-making (2). Therefore, being a member of or participating in a terrorist organisation, threatening with terrorist attacks, recruiting, and financing terrorism are also considered terrorist offences.

### Definition of Psychopathology

In this paper we use the term psychopathology for mental disorders and its traits and symptoms and psychological problems as stated in the DSM-5 (30, 49, 50). This definition highly resembles the definition of developmental psychopathology in youth (42–44). We do not use vulnerability as a concept related to mental health, because it is ill defined, and blurs the lines between mental health and risks of radicalisation or terrorism (51).

### Subjects

For this study we extracted a sample from the EDT of persons convicted of Jihadi terrorism between 2012 and 2021. Next we divided the sample based on two different age groups. The juvenile terrorist offender group consists of 46 young terrorist offenders between 15 and 21 years old at the time of their terrorist offence with a mean age of 19 (SD = 1.7). The adult terrorist offender group consists of 120 terrorist offenders, aged between 22 and 60 years with a mean age of 29 years (SD = 5.7).

Although there is ‘no bright line for defining youth’ and different upper limits and arguments are used (8, 52, 53), we choose to adhere to the definitions used in criminal law, since we studied convicted terrorists. In juvenile criminal law the age limit is 18 years although most European countries allow for young adults, aged 18–21 years, to be treated differently under criminal law than adults older than 21 years, either by sentencing them under juvenile criminal law or giving leniency under adult criminal law (54, 55). Following this line of reasoning we chose 21 years of age as an upper limit.

The group of 46 young terrorist offenders has 7 female terrorist offenders (15%), which is significantly more than in the older group (15 and 4%; *p* = 0.039), although the association is weak (Cramer's *V* = 0.189; df = 1). In the young offender group at least two-third have a migration background, comparable with the adult offender group. Almost half of the young terrorist offender group (44%) have a criminal record prior to their terrorist offence (one third with a violent offence), significantly less than the older group in which 64% committed one or more crimes (*p* = 0.022) although this association is also weak (Cramer's *V* = 0.180; df = 1). Except for gender and criminal history no other significant group differences were found (see Table 1)^1^.

**Table 1:** Background characteristics terrorist offenders by age group.

**Age groups**		**≤21 years (*****N*** **=** **46)**		**≥22 years (*****N*** **=** **120)**					
		**M**	**SD**	**M**	**SD**				
Age		19.3	1.7	28.6	5.7				
		***N***	**%**	**N**	**%**	***p*** ^*^	***V*** ^**^	**Odds-ratio**	**95% CI**
Gender	Male	39	84.8	113	94.2	**0.039**	0.189	0.247	0.254; 0.762
	Female	7	15.2	5	4.2				
	Missing	0	0	1	0.8				
Migration background	No	11	23.9	95	79.2	0.274	0.086	1.583	0.687; 3.651
	Yes	33	71.7	115	95.8				
	Missing	2	4.3	5	4.2				
Former crimes	No	24	52.2	41	34.2	**0.022**	0.180	2.254	1.115; 4.557
	Yes	20	43.5	77	64.2				
	Missing	2	4.3	2	1.7				

* *Fisher's exact test; p = 2-tailed*.

^*^p ≤ 0.05 identifies statistically significant differences between the two age groups (in bold).

** *V = Cramer's V*.

In the young terrorist offender group, 45 persons were convicted, one person died because of the terrorist act. In the older group 112 persons were convicted, eight persons died as a consequence of their terrorist act. Most of the young terrorist offenders (35%) were convicted for participating in a terrorist organisation, preparation (30%) or for membership of a terrorist organisation (26%). Compared to the older group, a significant higher percentage of the younger terrorists was involved in preparation (30 vs. 9%; *p* = 0.001; *V* = 0.266; df = 1) or incitement (22 vs. 5%; *p* = 0.002; *V* = 0.254; df = 1), indicating weak associations. Almost one-third of young offenders committed at least one of their terrorist offences alone, which is comparable with the older group (see Table 2). In both offender groups most of the subjects received unconditional prison sentences ranging from <3 months up until 10 years in the young offender group and 11 up until 15 years in the older group.

**Table 2:** Terrorist offence and criminal sentence by age group.

**Age groups**		**≤21 (*****N*** **=** **46)**		**≥22 (*****N*** **=** **120)**						
		***N***	**%**	***N***	**%**	***p*** ^*^	***V*** ^**^	**OR** ^***^	**95% CI**
Most committed terrorist offences (>1 possible)	Participating terrorist organisation	16	34.8	50	41.7	0.480	0.063	1.339	0.660; 2.716
	Preparation	14	30.4	11	9.2	**0.001**	0.266	0.231	0.095; 0.558
	Member terrorist organisation	12	26.1	28	23.3	0.691	0.029	0.862	0.394; 1.886
	Incitement	10	21.7	6	5.0	**0.002**	0.254	0.189	0.064; 0.558
	Financing	6	13.0	13	10.8	0.786	0.031	0.810	0.288; 2.276
	Recruiting	5	10.9	12	10.0	1.000	0.013	0.911	0.302; 2.747
	Supporting	5	10.9	13	10.8	1.000	0.995	0.996	0.334; 2.970
	Training	4	8.7	6	5.0	0.466	0.070	0.553	0.149; 2.056
Involvement other persons in offences^****^	No	12	26.1	34	28.3	0.848	0.021	0.898	0.415; 1.943
	Yes	33	71.7	84	70.0				
	Missing	1	2.2	2	1.7				
Most severe type of sentence	Unconditional prison sentence	43	93.5	104	86.7	0.401	0.156	–	
	Conditional prison sentence	2	4.3	3	2.5				
	Other	0	1.0	4	3.3				
	No sentence (subject died)	1	2.2	8	6.7				
	Missing	0	0.0	1	0.8				
Duration unconditional prison sentence (*n* = 147)	<3 months	3	7.0	4	3.8	0.258	0.278	–	
	3–5 months	4	9.3	2	1.9				
	6–11 months	2	4.7	8	7.7				
	1–2 years	18	41.9	28	26.9				
	3–4 years	9	20.9	30	28.8				
	5–6 years	4	9.3	15	14.4				
	7–10 years	2	4.7	7	6.7				
	11–15 years	0	0.0	5	4.8				
	Undefined period	1	2.3	4	3.8				
	Missing	0	0.0	1	1.0				

* *Fisher's exact test for 2 × 2 tables; Pearson Chi-Square test for 2 × >2 tables; p = 2-tailed*.

*p ≤ 0.05 identifies statistically significant differences between the two age groups (in bold).

** *Cramer's V*.

*** *OR = Odds Ratio*.

**** *Subject planned and/or executed the terrorist crime with other persons*.

## Data Sources

The dataset for the current study is exported from the EDT, which contains developmental, individual, and contextual potential risk factors for engagement in terrorist acts (10). The coded EDT data originates from primary source information from the police, public prosecution, court files, Ministry of Justice and prison administration, and probation reports from judicial institutions of the Netherlands, Belgium, six German federal states, Austria, and Sweden. The Netherlands and Belgium are overrepresented since they have more terrorist cases than Sweden and the six German federal states.

The EDT contains also extensive forensic mental health reports made by forensic psychiatrists and psychologists. The Dutch reports are pre-trial mental health reports for the court. These forensic reports include the history of psychosocial functioning, diagnostic assessments of psychopathology, DSM-5 classifications, the role of psychopathology in committing the terrorist acts, and advice on future risk and risk management. Forensic psychiatrists and psychologists receive professional feedback and follow diagnostic standards and guidelines. The forensic reports of the other participating Member States concern diagnostic assessments and advice in court for conditional release. These reports include personal and social history, psychosocial functioning, and advice on risk and risk management. Furthermore, they mention the role of psychopathology in committing terrorist acts. The methodological aspects of the forensic reports are comparable across the different Member States, due to standardised clinical assessments, the use of questionnaires for DSM 5 symptomatology and standardised risk assessments. This information was used by the researchers of the EDT. When in doubt, contact with the principal investigators was possible. Researchers could use a free space for comments for the indicators.

The formally allowed access for the judicial case file research of convicted terrorists is granted to one or two appointed researchers per Member State with the required expertise. They code all factors in a standardised way in the EDT, including the diagnostic symptoms and traits of mental disorders of forensic mental health reports (see “Variables”).

### Security and Privacy

Since personal data are processed in the EDT, research privacy and security measures are important considerations. The EDT has strict security requirements for data processing. Collected data by European researchers are directly entered and stored in the EDT, and all personal data are encrypted (10).

### Variables

For this study, we selected variables from different EDT domains (see Table 3). Gender, age at the time of the terrorist offence, and migrant status (subject lives temporary or permanently in a country where he or she was not born) originate from the domain “Demographic data”. The variable “Former crimes” was derived from the domain “Criminal history”. This item is coded if subject has a former police or judicial record, before the index crime. From the domain “Aspects crime” we selected the type of terrorist crime, the type of sentence, the length of prison sentence, and the involvement with other persons in the terrorist offence. This last variable was described as: “from judicial file information could be derived that other persons were involved in the index crime and/or named in the file, regardless of whether persons involved in the index crime are all part of same criminal/terrorist group and if the other persons are convicted or not. For example: in case subject is directed by a terrorist organisation how to execute the crime, although the crime itself can be executed alone”.

**Table 3:** EDT coding domains.

**Domains (number of items)**			
1	Compilation case file (7)	9	Prior to crime: personal acts (12)
2	Demographic data (58)	10	Preoccupation with weapons and violence (2)
3	Aspects crime (63)	11	Radicalization /ideology (15)
4	Criminal history (8)	12	Beliefs and attitudes (16)^*^
5	Personal history (47)	13	Social context and intention (22)*
6	Personality traits (30)	14	History, action and capacity (11)*
7	Psychiatric symptoms (35)	15	Commitment and motivation (18)*
8	Prior to crime: incidents (9)	16	Protection and risk mitigation (25)*

* *VERA-2R Domains*.

Mental disorders and its traits and symptoms were based on the extensive forensic mental health assessments. In the EDT, mental disorders and its traits and symptoms are operationalised in the domains: “Personality disorder and Traits” (PT) and “Other Psychiatric disorder and Symptoms” (PS) derived from the Diagnostic and Statistical Manual of Mental Disorders (49). The PT domain contains 10 DSM-5 personality disorders and their traits, the PS domain 10 DSM-5 mental disorders with underlying symptoms. In the EDT, a mental disorder can be coded as well as traits and symptoms if these are diagnosed in the forensic mental health report. In case traits or symptoms were mentioned not related to a corresponding mental disorder, coders could enter a separate item “other traits” or “other symptoms”, which was checked and recoded by the first author afterwards. Co-occurrence was coded in case of the prevalence of different mental disorders, including the underlying traits or symptoms (10).

Violent ideology, grievances and the anger about it were based on three VERA-2R indicators. We selected these three interrelated indicators because they are pertinent to extremist and terrorist beliefs (7, 56–58). The explanations of the indicators in the VERA-2R were copied into the EDT codebook and copied into the descriptions of the corresponding items in the EDT. The first item ‘Commitment to ideology that justifies violence” is described as: “Subject supports any ideology (political, religious, social or other cause) that justifies the use of violence to achieve ideological goals”. The second item “perceived grievances about injustice” is explained as: ‘Subject expresses any grievances that he/she and/or groups with which he/she identifies, are more deprived, oppressed or persecuted than they should be. The perceived grievances are related to political, religious, social or other issues. They do not have to be objectively true”. Finally, the “expressed emotions in response to perceived injustice” is described as “Any expression of anger, moral outrage or hatred in response to perceived injustice as individual or in terms of an identified group. Anger: Blaming or accusing people, threatening people, frightening people, or expressing feelings of revenge or vengeance. Moral outrage: Extreme passion and anger connected to a severe violation of moral principles. Hatred is a more intense emotion than anger or moral outrage.” In the EDT these indicators are operationalised for coding, specifying the low, moderate and high categories of the VERA-2R, and were coded based on the risk assessments of the forensic mental health and probation reports.

### Coding Procedure

The coding principle of the EDT is that researchers of participating European member states received a training to become familiar with the coding methodology and item definitions from the codebook. Inter-rater reliability (IRR) analyses of the training cases were calculated by measuring the percentage of agreement between each coder and the intended (golden standard) coding in five training cases. IRR analyses of the individual coders was done to diminish bias in coding between the different Member States due to various item interpretations.

Since the training cases had too little variation in the distributions of the ratings, kappa estimates appeared to be unrepresentatively low (59). Therefore, an alternative kappa was used, based on the percentage of agreement between the coders, and corrected for agreement based merely on chance, given the number of answer options. For the strength of the agreement, Landis and Koch's cut-off points were used (60). For items that are reported in this article, the IRR varied between 0.6 and 1.0. Feelings of anger as a personality trait had an IRR of 0.4, and should be interpreted with caution (see Table 4).

**Table 4:** Inter-rater reliability measures of included EDT items.

**EDT domain**	**EDT item**	IRR^*^
Demographic Data (DD)	Gender	1.00
	Migration background	0.96
Criminal History (CH)	Former crimes	1.00
Aspects Crime (CR)	Crime	0.79
	Involvement other persons	0.79
	Duration sentence	0.87
Psychiatric disorder and symptoms (PS)	Depressive symptoms (disorder)	0.83
	Intellectual disability disorder	0.78
	Psychotic/schizophrenic symptoms (disorder)	0.83
	Substance use symptoms (disorder)	0.86
	PTSD symptoms (disorder)	0.85
	Autism spectrum symptoms (disorder)	0.83
	Attention deficit hyperactivity symptoms (disorder)	0.77
	Anxiety symptoms (disorder)	0.65
	Neurocognitive disorder	0.88
Personality disorder and traits (PT)	Paranoid	0.90
	Schizoid	0.90
	Schizotypical	0.87
	Antisocial	0.85
	Borderline	0.86
	Histrionic	0.90
	Narcissistic	0.90
	Avoidant	0.90
	Dependent	0.91
	Obsessive-compulsive	0.85
	Problems with relationships	0.68
	Poor regulation of aggression	0.60
	Distrust or paranoid feelings	0.64
	Feelings of anger	0.44
Believes and Attitudes (BA)	Supports violent ideology	0.92
	Expressed grievances about perceived injustice	0.83
	Expressed anger in response to perceived injustice	0.74

* *Inter-rater reliability (IRR): based on the percentage agreement between each coder and “golden standard”, corrected for agreement based on chance given the number of answer options. The average is based on a sample of seven coders. “Feelings of anger” should be interpreted with caution due to a low IRR*.

### Statistical Analysis

All analyses for this study were carried out using IBM SPSS Statistics Version 26.0. To obtain more power in group comparisons, we dichotomized Likert scales scores for all included items. This resulted in two new categories: recoding into 0 of coding 0 (No, documented) and 1 (No, unlikely), and recoding into 1 for coding 2 (Yes, likely) and 3 (Yes, Documented). For the variables “grievances about perceived injustice” and “expressed emotions in response to perceived injustice” coding −99 (information fails) and coding 0 (not mentioned) were taken together. These factors were analysed on the basis of a forensic mental health report. If these factors were not mentioned by the forensic expert, it is assumed that they were not present. The average of the missing values on these items is similar in both age groups, 31% for the younger and 33% for the older terrorist offender group. As all included variables were measured at a nominal level after dichotomization and each observation was independent, data met the assumptions for a chi-square test. We conducted Pearson Chi-Square tests to compare the items and prevalence and type of psychopathology between age groups. For analyses with an expected number of <5 cases, we used Fisher's exact tests. Since our research aim was examining the association between psychopathology and risk factors in juvenile and older terrorist offender groups, for age group comparisons, and interactions of psychopathology with risk factors two-sided *p*-values were used, and alpha <0.05. To measure the strength of the associations between the risk factors, age groups and psychopathology, Cramer's *V* was used. This is an alternative to phi in 2 × 2 tables. To calculate Cramer's *V* the chi-square value is divided by the sample size, followed by the square root of this value. Similar to Pearson's r, a value close to 0 indicates no association. Values <0.3 indicate a weak association, 0.3–0.5 a moderate association and >0.5 indicating a strong association.

## Results

### Psychopathology

Thirty-one young terrorist offenders had a forensic mental health report from psychiatrists and/or psychologists (67%) and 75 older terrorist offenders (63%). A mental disorder was reported in about half of the persons in each age group. Psychopathology or clinically relevant traits and symptoms of a mental disorder were found in 81% of the young terrorist offenders and 73% of the adult terrorist offenders. This means that psychopathology (or clinically relevant traits and symptoms of a mental disorder) is 1.5 more prevalent in both age groups than mental disorders alone (see Tables 5a,b). Furthermore, psychopathology was not only found in lone acting terrorist offenders, but also among a selection of the offenders who committed their crime with others: when a mental health report was available, psychopathology was reported in 77% of the young offenders with group crimes and 69% of the adult offenders with group crimes, which was not significantly different. Interestingly, 88% of the young terrorist offenders and 70% of the adult terrorist offenders with a mental disorder were also diagnosed with a clinically relevant specific personality trait, not specifically linked to a personality disorder.

**Table 5a:** Psychopathology by age group^a^.

**Age groups**	**≤21 yrs (*****N*** **=** **31)**		**≥22 yrs (*****N*** **=** **75)**					
	***N***	**%**	***N***	**%**	p^*^	V^**^	OR^***^	**95% CI**
Psychopathology	25	80.6	55	73.3	0.470	0.077	0.660	0.236; 1.844
**Type of psychopathology**								
Depressive symptoms	7	22.6	14	18.7	0.789	0.045	0.787	0.283; 2.188
Intellectual disability	7	22.6	9	12	0.232	0.134	0.468	0.157; 1.394
Psychotic/schizophrenic symptoms	5	16.1	12	16	1.000	0.002	0.990	0.317; 3.094
Substance use symptoms	4	12.9	20	26.7	0.201	0.150	2.455	0.763; 7.894
PTSD symptoms	3	9.7	7	9.3	1.000	0.005	0.961	0.232; 3.984
Autism spectrum disorder symptoms	2	6.5	2	2.7	0.579	0.090	0.397	0.053; 2.955
ADHD symptoms	2	6.5	3	4	0.628	0.053	0.604	0.096; 3.806
Anxiety symptoms	2	6.5	5	6.7	1.000	0.004	1.036	0.19; 5.647
Neurocognitive problems	0	0	2	2.7	1.000	0.089	−	−
Cluster A personality traits	4	12.9	6	8	0.474	0.076	0.587	0.154; 2.244
Cluster B personality traits	10	32.3	31	41.3	0.511	0.085	1.480	0.612; 3.575
Cluster C personality traits	4	12.9	8	10.7	0.743	0.032	0.806	0.224; 2.901
**Specific personality traits (*****N*** **>** **1)**								
Problems with relationships	13	41.9	24	32.0	0.374	0.095	0.652	0.275; 1.544
Poor regulation of aggression	11	35.5	15	20.0	0.135	0.164	0.455	0.18; 1.15
Distrust or paranoid feelings	7	22.6	15	20.0	0.795	0.029	0.857	0.311; 2.364
Feelings of anger^****^	8	25.8	13	17.3	0.422	0.097	0.603	0.221; 1.642
Poor self-esteem	3	9.7	3	4.0	0.355	0.112	0.389	0.074; 2.043
Identity problems	2	6.5	4	5.3	1.000	0.022	0.817	0.142; 4.708
Co-occurrence psychopathology	20	64.5	49	65.3	1.000	0.008	1.037	0.432; 2.489

* *Fisher's exact test; p = 2-tailed*.

^*^p ≤ 0.05 significant difference between age groups.

** *Cramer's V*.

*** *OR, Odds Ratio*.

**** *Feelings of Anger has a low inter-rater reliability. Therefore, interpretation of this result have to be done with caution*.

a *Data from forensic mental health reports*.

**Table 5b:** Mental disorders by age group^a^.

**Age groups**	**≤21 yrs (*****N*** **=** **31)**		**≥22 yrs (*****N*** **=** **75)**					
	***N***	**%**	***N***	**%**	p^*^	V^**^	**Odds-ratio**	**95% CI**
Mental disorder	16	51.6	33	44	0.525	0.069	0.737	0.318; 1.705
Type of mental disorder								
Depressive disorder	3	9.7	4	5.3	0.415	0.08	0.526	0.111; 2.501
Intellectual disability disorder	7	22.6	9	12	0.232	0.134	0.468	0.157; 1.394
Psychotic/schizophrenic disorder	3	9.7	7	9.3	1.000	0.005	0.961	0.232; 3.984
Substance use disorder	2	6.5	12	16	0.226	0.128	2.762	0.58; 13.146
PTSD	0	0	2	2.7	1.000	0.089	–	–
Autism spectrum disorder	1	3.2	1	1.3	0.501	0.063	0.405	0.025; 6.694
ADHD	2	6.5	1	1.3	0.204	0.14	0.196	0.017; 2.245
Anxiety disorder	1	3.2	1	1.3	0.501	0.063	0.405	0.025; 6.694
Neurocognitive disorder	0	0	2	2.7	1.000	0.089	–	–
Cluster A personality disorder	1	3.2	1	1.3	0.501	0.063	0.405	0.025; 6.694
Cluster B personality disorder	4	12.9	6	8	0.474	0.076	0.587	0.154; 2.244
Cluster C personality disorder	1	3.2	1	1.3	0.501	0.063	0.405	0.025; 6.694

* *Fisher's exact test; p = 2-tailed; p ≤ 0.05 statistically significant differences between two age groups*.

** *Cramer's V*.

a *Data from forensic mental health reports*.

The prevalence of psychopathology is comparable in both groups. Problems with relationships is the most mentioned personality trait in both groups (42% in the young group and 32% in the adult group). Poor regulation of aggression, distrust or paranoid feelings, and feelings of anger are also relatively often reported in both groups, with a prevalence between 23 and 36% in the young age group and from 17% till 20% in the adult group. Cluster B personality disorder and/or traits (32%), a depressive disorder and/or symptoms (23%), and an intellectual disability disorder (23%) were most reported in the young terrorist offender group, followed by a psychotic or schizophrenic disorder and/or symptoms (16%), a substance use disorder and/or symptoms (13%), and cluster A or C personality disorder and/or traits (both 13%). Co-occurrence of psychopathology was reported in 65% (*N* = 20) of the 31 young terrorist offenders with a mental health report, which is comparable with the older group.

### Violent Ideology, Grievances, and Anger About Perceived Injustice

Almost all of the young terrorist offenders supported an ideology that justifies the use of violence (97%). More than half of the young terrorist offenders expressed grievances about perceived injustice prior to their terrorist offence (65%). Furthermore, a third expressed anger about perceived injustice (36%). Comparisons between both age groups showed no significant differences (see Table 6). The most commonly reported perceived general grievances by 12 young and 18 adult terrorist offenders concern the feeling that Muslims are discriminated by Western society. The most commonly perceived personal grievances by 10 young and 22 adult terrorist offenders mentioned also Muslim discrimination.

**Table 6:** Ideology, grievances, and anger by age group^a^.

**Age groups**		**≤21 yrs (*****N*** **=** **31)**		**≥22 yrs (*****N*** **=** **75)**				
		***N***	**%**	***N***	**%**	p^*^	V^**^	**Odds-ratio**	**95% CI**
Supports violent ideology	No	1	3.2	4	5.3	1.000	0.058	0.525	0.056; 4.902
	Yes	30	96.8	63	84.0				
	Missing	0	0.0	8	10.7				
Expressed grievances about perceived injustice	No	11	35.5	39	52.0	0.139	0.151	0.508	0.214; 1.205
	Yes	20	64.5	36	48.0				
Expressed anger in response to perceived injustice	No	20	64.5	50	66.7	0.826	0.021	0.909	0.378; 2.188
	Yes	11	35.5	25	33.3				

* *Fisher's exact test; p = 2-tailed; p ≤ 0.05 identifies statistically significant differences between two age groups*.

** *Cramer's V*.

a *Data from forensic mental health reports*.

### Psychopathology and Violent Ideology, Grievances, and Anger About Perceived Injustice

Despite the high prevalence of a violent ideology, grievances of perceived injustice and the anger about it, no significant associations were found with psychopathology in the young terrorist offender group (see Table 7a). In the older group, psychopathology could be linked to expressed grievances (*p* = 0.020, Cramer's *V* = 0.278, df = 1) and to the expressed anger about it (*p* = 0.012, Cramer's *V* = 0.298, df = 1), both indicating small effects (61). See Table 7b.

**Table 7a:** Psychopathology and ideology, grievances, and anger in young age group ≤21 years^a^.

**Psychopathology**	**No**		**Yes**			
	***N***	**%**	***N***	**%**	p^*^	V^**^	**Odds-ratio**	**95% CI**
Supports violent ideology	6	100	24	96	1.000	0.089	1.250	1.045; 1.495
Expressed grievances about perceived injustice	2	33.3	18	72	0.151	0.319	5.143	0.763; 34.686
Expressed anger in response to perceived injustice	2	33.3	9	36	1.000	0.022	1.125	0.171; 7.399

* *Fisher's exact test; p = 2-tailed; p ≤ 0.05 identifies statistically significant differences between the two age groups*.

** *Cramer's V*.

a *Data from forensic mental health reports*.

**Table 7b:** Psychopathology and ideology, grievances, and anger in adult age group ≥22 years^a^.

**Psychopathology**	**No**		**Yes**			
	***N***	**%**	***N***	**%**	p^*^	V^**^	**Odds-ratio**	**95% CI**
Supports violent ideology	16	94.1	47	94.0	1.000	0.002	0.979	0.095; 10.096
Expressed grievances about perceived injustice	5	25.0	31	56.4	**0.020**	0.278	3.875	1.235; 12.163
Expressed anger in response to perceived injustice	2	10.0	23	41.8	**0.012**	0.298	6.469	1.365; 30.661

* *Fisher's exact test; p = 2-tailed; p ≤ 0.05 identifies statistically significant differences between the two age groups (in bold)*.

** *Cramer's V*.

a *Data from forensic mental health reports*.

Although in the young terrorist group no association could be found between grievances about perceived injustice and having any psychopathology, a positive and moderate association (*p* = 0.033; Cramer's *V* = 0.401, df = 1) was found between grievances and in depressive symptoms. We did not find this the older group (see Tables 8a,b).

**Table 8a:** Ideology, grievances, anger and depressive symptoms in young age group ≤ 21 years^a^.

**Depressive symptoms**	**No**		**Yes**					
**Ideological indicator**	***N***	**%**	***N***	**%**	p^*^	V^**^	**OR**	**95% CI**
Supports violent ideology	23	95.8	7	100	1	0.099	–	
Expressed grievances about perceived injustice	13	54.2	7	100	0.033	0.401	–	
Expressed anger in response to perceived injustice	6	25.0	5	71.4	0.067	0.406	7.5	1.142; 49.26

* *Fisher's exact test; p ≤ 0.05 identifies statistically significant differences between the two age groups (p=2-tailed)*.

** *Cramer's V. OR, Odds Ratio*.

a *Selection of persons with a forensic mental health report*.

**Table 8b:** Ideology, grievances, anger and depressive symptoms in adult age group ≥22 years^a^.

**Depressive symptoms**	**No**		**Yes**					
**Ideological indicator**	***N***	**%**	***N***	**%**	p^*^	V^**^	**OR**	**95% CI**
Supports violent ideology	52	92.9	11	100	1.000	–	–	
Expressed grievances about perceived injustice	30	49.2	6	42.9	0.771	0.049	0.775	0.240; 2.501
Expressed anger in response to perceived injustice	21	34.4	4	28.6	0.763	0.048	0.762	0.213; 2.724

* *Fisher's exact test; p ≤ 0.05 identifies statistically significant differences between the two age groups (p=2-tailed)*.

** *Cramer's V. OR, Odds Ratio*.

a *Selection of persons with a forensic mental health report*.

In the adult terrorist group we found a positive but weak association (*p* = 0.020, Cramer's *V* = 0.268, df = 1) between expressed anger about perceived injustice and cluster B personality disorder or traits. We did not find this in the young group (see Tables 8c,d).

Finally, a significant and strong association (*p* = 0.000, Cramer's *V* = 0.630, df = 1) was present between grievances about perceived injustice and problems with relationships in the young group (see Table 8e). In the adult group a positive association was also found between problems with relationships and these grievances (*p* = 0.026, Cramer's *V* = 0.256, df = 1) but also with anger in response to perceived injustice (*p* = 0.036, Cramer's *V* = 0.243, df = 1). See Table 8f.

**Table 8c:** Ideology, grievances, anger and cluster b personality traits in young age group ≤21 years^a^.

**Cluster B personality traits**	**No**		**Yes**					
**Ideological indicator**	***N***	**%**	***N***	**%**	p^*^	V^**^	**OR**	**95% CI**
Supports violent ideology	20	95.2	10	100	1.000	0.126	–	–
Expressed grievances about perceived injustice	11	52.4	9	90.0	0.055	0.368	8.182	0.874; 76.58
Expressed anger in response to perceived injustice	9	42.9	2	20.0	0.262	0.223	0.333	0.030; 2.966

* *Fisher's exact test; p ≤ 0.05 identifies statistically significant differences between the two age groups (p = 2-tailed)*.

** *Cramer's V. OR, Odds Ratio*.

a *Selection of persons with a forensic mental health report*.

**Table 8d:** Ideology, grievances, anger and cluster b personality traits in adult age group ≥22 years^a^.

**Cluster B personality traits**	**No**		**Yes**					
**Ideological indicator**	***N***	**%**	***N***	**%**	p^*^	V^**^	**OR**	**95% CI**
Supports violent ideology	36	94.7	27	93.1	1.000	0.034	0.75	0.099; 5.668
Expressed grievances about perceived injustice	17	38.6	19	61.3	0.053	0.223	2.515	0.979; 6.461
Expressed anger in response to perceived injustice	10	22.7	15	48.4	0.020	0.268	3.188	1.177; 8.636

* *Fisher's exact test; p ≤ 0.05 identifies statistically significant differences between the two age groups (p = 2-tailed)*.

** *Cramer's V. OR, Odds Ratio*.

a *Selection of persons with a forensic mental health report*.

**Table 8e:** Ideology, grievances, anger and problems with relationships in young age group ≤21 years^a^.

**Problems with relationships**
	**No**		**Yes**				
**Ideological Indicator**	***N***	**%**	***N***	**%**	p^*^	V^**^	**OR**	**95% CI**
Supports violent ideology	18	100	12	92.3	0.419	0.215	-	
Expressed grievances about perceived injustice	7	38.9	13	100	0.000	0.630	2.857	1.572; 5.192
Expressed anger in response to perceived injustice	5	27.8	6	46.2	0.449	0.190	2.229	0.497; 9.997

* *Fisher's exact test; p ≤ 0.05 identifies statistically significant differences between the two age groups (p = 2-tailed)*.

** *Cramer's V. OR, Odds Ratio*.

a *Selection of persons with a forensic mental health report*.

**Table 8f:** Ideology, grievances, anger and problems with relationships in age group ≥22 years^a^.

**Problems with relationships**	**No**		**Yes**					
**Ideological indicator**	***N***	**%**	***N***	**%**	p^*^	V^**^	**OR**	**95% CI**
Supports violent ideology	41	93.2	22	95.7	1.000	0.050	1.610	0.158; 16.408
Expressed grievances about perceived injustice	20	39.2	16	66.7	0.046	0.256	3.100	1.120; 8.579
Expressed anger in response to perceived injustice	13	25.5	12	50.0	0.036	0.243	2.923	1.056; 8.092

* *Fisher's exact test; p ≤ 0.05 identifies statistically significant differences between the two age groups (p = 2-tailed)*.

** *Cramer's V. OR, Odds Ratio*.

a *Selection of persons with a forensic mental health report*.

## Discussion

Several studies revealed the prevalence of mental disorders among terrorist offenders (27–29). We examined the extent to which psychopathology is present in young terrorist offenders and hypothesised that psychopathology might be associated with ideological risk factors. Moreover, we hypothesised that these associations differed for young and adult terrorist offenders.

We found that most adult and young Jihadist terrorist offenders with a forensic mental health report had psychopathological problems. Most frequently found were symptoms and traits of intellectual disability disorders, depressive disorders, psychotic/schizophrenic disorders, substance disorders, and personality disorders. Most frequently found clinically relevant personality traits were problems with relationships, poor regulation of aggression, feelings of anger, and paranoid feelings. More important, we found some first indications for a positive association between psychopathology and grievances, and anger about perceived injustice. In the young terrorist offenders with depressive symptoms, grievances about perceived injustice were more often present than in young terrorist offenders without these symptoms. In adult terrorist offenders it was found that grievances about perceived injustice and the anger were related to cluster B personality traits (narcissistic and antisocial personality traits). In addition, in both young and adult terrorist offenders expressed grievances about perceived injustice was related to problems with relationships.

The association of depressive symptoms and grievances about perceived injustice in the group of young terrorist offenders can be understood when this is compared to young violent offenders. Research shows that compared to adults, young violent offenders have higher rates of depression and developmental disorders (12).

The association of emotions related to perceived injustice and personality disorders and traits is not surprising. Anger about perceived injustice and a hostile attribution style are also symptoms and traits of psychopathology in antisocial youth, in personality disorders, and in psychosis (45, 46). Therefore, these personality traits may be interrelated with perceived injustice and as a consequence may make individuals more prone to terrorism.

The associations between psychopathology and anger about perceived injustice were not found in young terrorist offenders. This may be explained by the fact that our group of young terrorist offenders was small. Moreover, the adult group of terrorist offenders contained relatively young people of 22 years and older, with a mean age of 29 years. Set against brain development of areas important in offending (43, 62) future research should focus on larger groups but may also consider comparisons of adolescents, young adults, and older offenders.

The associations between grievances about perceived injustice and problems with relationships in the young and older terrorist offender group might be due to a lack of empathy or little understanding for people outside the ideological group with an us-and-them perspective. This can also lead to information being withheld from forensic mental health experts about motives or ideology.

In addition, more than 75% of both young and adult terrorist offenders who were diagnosed with a mental health disorder also met criteria for clinically relevant other personality traits, e.g., poor regulation of aggression, feelings of anger, distrust or paranoid feelings or problems with relationships. Since our results showed a positive association of this last trait with grievances or anger to perceived injustice, it highlights the significance to not only focus on mental disorders, but also on its clinically relevant traits and symptoms.

Finally, we found psychopathological problems in young and adult terrorist offenders who acted alone and in a group, in contrast to findings of other studies (27). This might be due to our definition of psychopathology in which we also include traits and symptoms. Future studies could focus on the differences in psychopathology between terrorists acting alone and in groups, taking into account the symptoms and characteristics of mental disorders.

This study has given preliminary results on psychopathology and its association with violent ideology, grievances, and anger about perceived injustice among a sample of European adult and young Jihadist terrorist offenders. Although the study is based on thorough judicial data including the standardised coding of psychopathology of forensic mental health assessments, there are some limitations.

First, the results are based on small sample sizes and therefore must be treated with caution. We need more data to improve statistical power. With a developing EDT with different European member states, the number of included cases of terrorist offenders will expand in the near future.

Second, selection bias can be an issue in a small sample. Dutch and Belgian cases are overrepresented in our sample of young terrorist offenders, and it remains to be seen whether these results can be extrapolated to young Jihadist terrorist offenders from other countries. This also applies to the results regarding psychopathology, since forensic mental health reports were available for two-thirds of the convicted young terrorist offenders in our sample.

Third, our results seems to be representative with respect to migration background, and criminal history, since similar percentages were found in other studies on European jihadists (63, 64). But questions remain about other terrorist offenders, although there might be notable differences across extremist ideologies, but also similarities (65). Questions also remain about differences between male and female terrorist offenders, for example in relation to terrorist offences, psychopathology and the interaction with risk indicators. We would like to describe this in a separate article.

Fourth, the results regarding psychopathology are based on a sample of 31 of the 46 young terrorist offenders for whom a forensic mental health report was available as part of the primary source information. In this sample 25 of the 31 the young offender group (81%) was diagnosed with any type of psychopathology. Since this sample comprises only terrorist offenders with a mental health report, consequently, no statements can be made about the exact prevalence of psychopathology among the total young terrorist offender group. However, we do know that from the total young terrorist offender group of 46 persons, 25 persons had a diagnosis of psychopathology. This seems to suggest a percentage of at least 50% of psychopathology in the total young offender group.

The most important strength of our study is the use of the unique dataset from the EDT. This enabled detailed research into psychopathology and background information of young and adult terrorist offenders and their offences, based on extensive judicial information and thorough forensic mental health reports. The enlarging dataset enables us to perform more detailed analyses, including the relation between psychopathology and different type of terrorist offences.

We are well aware that our results do not point to simple causal relationships and that associations between psychopathology and terrorist engagement can lead to stigmatisation, and media coverage about ‘bad’ and ‘mad’ paradigms (38, 66, 67) similar to what is seen with violent offences (68). Future empirical studies should correlate psychopathology with biological, developmental, and contextual risk factors as social and family bonds (online), networks, educational achievements, triggering events for terrorist acts, and type of terrorist involvement (8, 9, 40, 69) to give more in depth information on this matter to further our knowledge. This may possibly nuance discussions about ‘mad’ or ‘bad’ and lead to more effective interventions based on risks, needs and responsivity principles.

In sum, the first results on young terrorist offenders using data from the EDT may be relevant for forensic practise. Our results show the first indications for the association of psychopathology and grievances, and the anger about perceived injustice in young and older terrorist offenders. They also show the relevance to include psychopathology in risk assessment instruments such as the VERA-2R. In the near future, we will expand the number of terrorists included in our European database and will compare (young) terrorist offenders with a control group of regular (young) violent offenders in relation to psychopathology, contextual factors, group functioning and offending. This can possibly lead to more knowledge of sub-groups of terrorist offenders committing different type of terrorist acts, with potentially different clusters of risk indicators.

## Data Availability Statement

The dataset presented in this article are not readily available because we have strong privacy rules related to data sharing. Requests to access the dataset should be directed to d.alberda@dji.minjus.nl.

## Author Contributions

ND and DA contributed to the conception and design of the study. DA organised the database and performed the statistical analysis. ND wrote the first draft of the manuscript. All authors contributed to manuscript revision, read, and approved the submitted version.

## Funding

The development of the EDT was funded for 2 years by the EU from 2017 (JUST-AG-2016-03, project number 763765) and a continuation of the EDT from 2020 (JUST-JCOO-AG-2020, project number 101007383). The Dutch Counterterrorism Organisation and the Dutch ministry of Justice and Safety funded the EDT partly in 2019, 2020, and 2021.

## Conflict of Interest

The authors declare that the research was conducted in the absence of any commercial or financial relationships that could be construed as a potential conflict of interest.

## Publisher's Note

All claims expressed in this article are solely those of the authors and do not necessarily represent those of their affiliated organizations, or those of the publisher, the editors and the reviewers. Any product that may be evaluated in this article, or claim that may be made by its manufacturer, is not guaranteed or endorsed by the publisher.

## Footnotes

^1^Nineteen persons were excluded because of missing age information.
